# Quantification of ortholog losses in insects and vertebrates

**DOI:** 10.1186/gb-2007-8-11-r242

**Published:** 2007-11-16

**Authors:** Stefan Wyder, Evgenia V Kriventseva, Reinhard Schröder, Tatsuhiko Kadowaki, Evgeny M Zdobnov

**Affiliations:** 1 Department of Genetic Medicine and Development, University of Geneva Medical School, 1211 Geneva, Switzerland; 2 Swiss Institute of Bioinformatics, rue Michel-Servet, 1211 Geneva, Switzerland; 3 Department of Structural Biology and Bioinformatics, University of Geneva Medical School, rue Michel-Servet, 1211 Geneva, Switzerland; 4 Interf. Institut für Zellbiologie, Abt. Genetik der Tiere, Universität Tübingen, 72076 Tübingen, Germany; 5 Graduate School of Bioagricultural Sciences, Nagoya University, Chikusa, Nagoya 464-8601, Japan; 6 Imperial College London, South Kensington Campus, London SW7 2AZ, UK

## Abstract

**Background:**

The increasing number of sequenced insect and vertebrate genomes of variable divergence enables refined comparative analyses to quantify the major modes of animal genome evolution and allows tracing of gene genealogy (orthology) and pinpointing of gene extinctions (losses), which can reveal lineage-specific traits.

**Results:**

To consistently quantify losses of orthologous groups of genes, we compared the gene repertoires of five vertebrates and five insects, including honeybee and *Tribolium *beetle, that represent insect orders outside the previously sequenced Diptera. We found hundreds of lost Urbilateria genes in each of the lineages and assessed their phylogenetic origin. The rate of losses correlates well with the species' rates of molecular evolution and radiation times, without distinction between insects and vertebrates, indicating their stochastic nature. Remarkably, this extends to the universal single-copy orthologs, losses of dozens of which have been tolerated in each species. Nevertheless, the propensity for loss differs substantially among genes, where roughly 20% of the orthologs have an 8-fold higher chance of becoming extinct. Extrapolation of our data also suggests that the Urbilateria genome contained more than 7,000 genes.

**Conclusion:**

Our results indicate that the seemingly higher number of observed gene losses in insects can be explained by their two- to three-fold higher evolutionary rate. Despite the profound effect of many losses on cellular machinery, overall, they seem to be guided by neutral evolution.

## Abstract 2

Comparison of the gene repertoires of 5 vertebrates and 5 insects showed that the rate of losses correlates well with the species' rates of molecular evolution and radiation times and suggests that the Urbilateria genome contained more than 7,000 genes.

## Background

The evolution of gene repertoires is mostly driven by gene duplications and gene losses. Duplications can arise by short-range copying of individual genes or of longer multigene DNA segments, or even result from whole genome duplications [1,2]. Copies of single genes are frequently associated with retrotransposition [3], whereas unequal homologous recombination copies DNA segments of varying length. Gene proliferation, on the other hand, is balanced by gene losses, either through acquiring deleterious mutations that eventually disable the genes or as a consequence of unequal homologous recombination.

Massive gene losses of olfactory receptors were reported in human and ape families compared to mice [4], which have been speculated to be linked with the acquisition of full trichromatic vision, lowering the demand for olfaction [5]. Ohlson's 'less-is-more' hypothesis emphasizes that loss-of-function mutations may play a beneficial role in evolution [6]; an example for adaptive gene loss is the near-complete fixation of a null allele of *CASP12 *in the human lineage [7], presumably to confer protection from severe sepsis. Gene losses have also been implicated in reproductive isolation of *Drosophila *races [8].

The fast growing number of available vertebrate and insect genomes allows increasingly refined comparisons and the quantification of the major modes of animal genome evolution. The recent sequencing of the honeybee [9] and the *Tribolium *beetle [10] genomes extends insect genomics from only Dipteran species to the orders of Hymenoptera and Coleoptera, which radiated about 300 million years ago [11]. This allowed us to quantify and date losses of orthologous groups across ten bilaterian species in the first analysis that consistently compares five insects (phylum Arthropoda) and five vertebrates (phylum Chordata). Previous studies of gene losses have been focussed on mammals [12], vertebrates [13], or included only a single insect [14], or two dipterans [15]. Reassuringly, our analysis recovered previously published cases of gene losses, such as the loss of DNA methylation [16], and the heavy rearrangement of the circadian clock [17] in Diptera.

## Results

### Quantification of ortholog losses

To systematically identify gene losses in vertebrate and insect representatives of Bilaterian species, we delineated orthologous groups based on all-against-all Smith-Waterman comparisons using the official gene sets of five vertebrates (human, mouse, opossum, chicken and pufferfish) and five insects (fruitfly, malaria mosquito, dengue/yellow fever mosquito, honeybee and red flour beetle) (see details in Materials and methods). The species choice aimed to maximize the phylogenetic coverage with similar lineage radiation times in both deuterostomia and protostomia. The fraction of genes with recognized orthology among these species represents about 70-80% of their predicted gene pools. The comparative analysis of the shared content of gene repertoires across these species is discussed in the study presenting the analysis of the first beetle genome, that of *Tribolium castaneum *[10], and here we focus on the analysis of losses of orthologous genes. According to their phyletic distribution and gene copy-number in each of the species, we considered the following types of orthologous groups reflecting different selection pressures: the universal single-copy orthologs (U); the universal multiple-copy orthologs (N); patchy orthologs (P) that are present in both phyla in at least three species, in single or multiple copies; and insect- or vertebrate-specific orthologs (I and V, respectively). While universal single-copy orthologs (U) are evolving under a distinct pressure for copy-number control, the number of universal multiple-copy orthologs (N) under similarly strict copy-number control is extremely low (only six groups have equal multiple-copy gene number in at least nine species). Although U, N and P orthologs must all have been present in the last common ancestor of insects and vertebrates, the Urbilateria, losses in these fractions occur at different rates.

**Figure 1:**
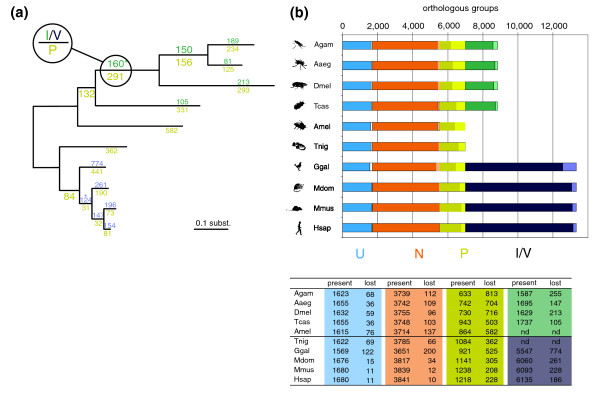
Quantification of orthologous gene losses in insects and vertebrates. **(a) **The phylogenetic relations among the organisms are illustrated by the tree, with branch length proportional to the rate of amino acid substitutions estimated using the maximum-likelihood approach. The number of orthologous groups lost on the internal phylogenetic branches were inferred using the Dollo parsimony principle and are shown on the phylogenetic tree above branches for the I/V fraction, and below branches for the P fraction. *The presence in two species was sufficient to infer losses of I/V orthologous groups. **(b) **The number of orthologous group losses in the five main categories: U, universal single-copy genes (blue, present in all species except the one in question); N, universal multiple-copy genes (orange, present in at least nine species); P, patchy orthologs (yellow, present in both phyla in at least three species, in one or multiple copies); I/V, insect- or vertebrate-specific orthologous groups (present only in insects (green) or vertebrates (violet), in at least three species. The dark parts of the bars depict the number of contemporarily present orthologous groups, and the light parts depict the number of inferred losses. AGAM, *Anopheles gambiae*; AAEG, *Aedes aegypti*; DMEL, *Drosophila melanogaster*; TCAS, *Tribolium castaneum*; AMEL, *Apis meliferia*; HSAP, *Homo sapiens*; MMUS, *Mus musculus*; MDOM, *Monodelphis domestica*; GGAL, *Gallus gallus*; TNIG, *Tetraodon nigroviridis*.

Figure 1b shows the size distribution of the orthologous fractions and the number of losses in each species and ortholog category. The tree shown in Figure 1a illustrates the species phylogeny, which allowed us to infer the number of losses on the internal branches, assuming evolutionary parsimony, which minimizes the number of events required to explain the phyletic gene distribution in each orthologous group. The phylogenetic tree was reconstructed using maximum-likelihood analysis of the concatenated alignment of 1,150 universal single-copy orthologs [10] where the lengths of the branches are proportional to the number of accumulated mutations, allowing us to compare the gene loss rates with the rates of lineage divergence (measured as the rate of protein substitutions). For the branches closest to the root, the numbers of gene losses cannot be inferred without an additional outgroup.

The analysis identified hundreds of differentially lost Urbilaterian genes of U, N and P orthologs in each of the species (see table of Figure 1). Overall, about 40% of ancient orthologous groups have been lost in at least one (out of the ten) species, illustrating the extent of the evolutionary flexibility of gene pools. Moreover, there are dozens of genes lost in each of the species that otherwise appear as universal single-copy orthologs. Koonin *et al*. [14] noted that nearly all pan-eukaryotic single-copy orthologs are subunits of known protein complexes; nevertheless, the observed losses indicate that even seemingly tightly constrained genes are, to a certain degree, dispensable in evolution.

### Number of losses correlate with molecular divergence

**Figure 2:**
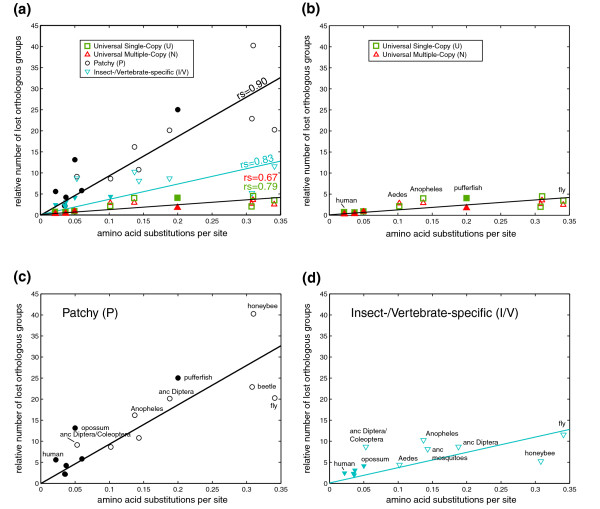
The number of ortholog losses correlates with the rate of amino acid substitutions. The number of orthologous group (U, N, P, I/V) losses normalized with the total size of the fraction is plotted versus the branch length of the maximum-likelihood phylogenetic tree (Figure 1). **(a) **All ortholog types combined; **(b) **U and N orthologs; **(c) **Patchy orthologs; **(d) **Insect- and vertebrate-specific orthologs. Filled symbols denote vertebrates and open symbols denote insects. Spearman rank correlations: U orthologs, rs = 0.79, *p *= 0.015; N orthologs, rs = 0.67, *p *= 0.05; P orthologs, rs = 0.90, *p *< 0.01; I/V orthologs, rs = 0.83, *p *< 0.01. Regression slopes for U and N are not statistically different. Anc, ancestral.

The inferred distribution of losses over the internal branches of the species phylogeny allowed us to correlate them with the estimated geological time of species radiations and the molecular evolutionary rate in each of the lineages. The molecular rates of evolution were quantified using genome-wide maximum-likelihood analysis of amino acid substitutions in the well aligned regions of single-copy orthologs (see Materials and methods). Figure 2a displays the correlation of the number of losses of the different types of orthologs plotted versus the protein sequence divergence. Losses of U and N orthologs (Figure 2b) occur only at the terminal branches as the fraction definition requires presence of the orthologous genes in at least nine species, whereas losses of P orthologs (Figure 2c) occur at both internal and terminal branches. Correlations are statistically significant for all categories (see Figure 2 legend for details), and there is no distinction between insects and vertebrates. Moreover, although the absolute numbers of insect- and vertebrate-specific losses appear different, they in fact follow the same trend when normalized to the total number of the phylum-specific orthologous groups (Figure 2d; see Additional data file 1 for a graph with absolute numbers). The different slopes of the regression lines reflect the varying stringency of evolutionary constraints that differ between the postulated types of orthologs. Despite the difference in the absolute numbers of lost U and N orthologs, their rates of loss are indistinguishable when normalized to the number of such orthologous groups, indicating the same level of purifying selection (Figure 2b). The data show that P orthologs are about 8-fold less constrained than U and N orthologs; this roughly corresponds to about 20% of the common Bilateria gene pool evolving 8 times faster than the remaining, more constrained fraction. I and V orthologs appear to be about three-fold more constrained than P orthologs, which is not surprising as they may represent a similar mixture of a slower evolving fraction of 80% and a faster evolving minority. The level of correlation between the number of losses and the protein sequence divergence rates (Figure 2) is similar to that observed between other genome-wide measures of species divergence [18]. Chicken was excluded from this and all following analyses as a clear outlier (see Discussion).

### Insects evolve two to three times faster than vertebrates

Protein sequence divergence is significantly larger between insects than between vertebrates (see the longer branch lengths in Figure 1; Mann-Whitney U test, *p *= 0.009). Similarly, this is reflected in the observation of significantly more frequent gene losses in insects than in vertebrates (Mann-Whitney U test: N orthologs, *p *= 0.016; P orthologs, *p *= 0.04). In comparison with vertebrates, the rate of evolution in bee and beetle is about two-fold higher and up to three-fold higher in Diptera. This especially high rate of evolution in Diptera, particularly at the base of the Dipteran radiation, has been noted previously [19].

### Lower estimate of the Urbilateria number of genes

**Figure 3:**
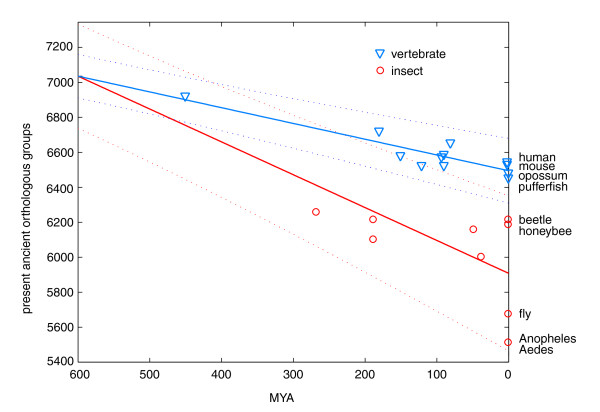
Extrapolation of number of ancient (U, N and P) orthologs to Urbilateria. The regression lines (and their 90% confidence intervals) are drawn using the number of U, N and P orthologous groups in current species, the estimates for putative ancestors, including the inferred number of losses (Figure 1), and the assumed split of insects and vertebrates about 600 MYA against the species radiation time. Remarkably, the naïve counting of orthologous groups that have at least one insect and at least one vertebrate member results in 7,114 likely Urbilateria genes.

Despite inherent dating uncertainties, the correlation between the number of lost orthologous groups and divergence times is significant for U and P orthologs (Spearman rank correlations: U orthologs, rs = 0.84, *p *= 0.007; N orthologs, rs = 0.58, *p *= 0.11; P orthologs, rs = 0.57, *p *= 0.03), indicating that losses of ancient genes occur in a roughly clock-like manner. The good correlation between the rate of losses with molecular rate and time indicates their stochastic nature. Projection of these trends as shown in Figure 3 to 600 million years ago (MYA), presumably dating the radiation of insects and vertebrates, suggests that over 1,000 (95% confidence interval 799-1,456) Urbilaterian genes have been lost from insects and only half this number (95% confidence interval 404-678) from vertebrates. This leads to the lower estimate of the number of Urbilaterian genes of just over 7,000 (remarkably, we obtained 7,114 orthologous groups with at least one insect and one vertebrate member). This estimate, however, does not take into account: genes that currently appear as insect- or vertebrate-specific, many of which could be of Urbilaterian origin; closely related Urbilateria paralogs that remain unresolved and are likely grouped together in N groups; as well as fractions of fast diverging genes that escaped our orthology classification.

### Functional load of losses

#### Recovered known facts as positive controls

Reassuringly, closer inspection of several of the predicted cases of lost genes pointed to recently published findings of lineage-specific biology.

##### Hedgehog signaling pathway rearrangements in *Drosophila*

Hedgehog signaling pathway rearrangements in *Drosophila *have been reported where orthologs of human *Sil*, *Hip *and *Gas1 *are missing from *Drosophila *[20], and homologs of polaris/TG737 (nompB) and *Kif3a *(*Klp64D*) appear to have roles unrelated to hedgehog signaling [21,22].

##### *Sid-1/tag-130 *gene loss in all Diptera

*Sid-1*/*tag-130 *genes have been lost in all Diptera but are present in bee and *Tribolium *as reported by Weinstock *et al*. [9]. *Sid-1 *is implicated in the cellular import of RNA interference signal and enables passive uptake of double-stranded RNA (yet, *Sid-1 *is likely to be a *Caenorhabditis elegans *invention as its inparalog, *TAG130*, is less derived (Additional data file 2)).

##### DNA-methyltransferases DNMT1 and DNMT3B lost in the Coleoptera/Diptera ancestor

DNA-methyltransferases DNMT1 and DNMT3B have been lost in the Coleoptera/Diptera ancestor, consistent with their loss reported in Diptera [23] and their surprising presence in honeybee [9,16].

##### A candidate insect telomerase reverse transcriptase

A candidate insect telomerase reverse transcriptase (TERT) is present in honeybee and *Tribolium *but absent in all Dipterans. The absence of TERT in Diptera seems to be correlated with the loss of telomeric TTAGG repeats [24].

##### Sterol metabolism, NAD biosynthesis, and other losses

Sterol metabolism, NAD biosynthesis, and other losses proposed earlier from the comparison of the fly and the mosquito genomes [23] seem to have been lost in all insects sequenced so far, that is, before the appearance of holometabolous insects. Exceptions are a dihydroxyacetone kinase 1 lost from both *Drosophila *and *Anopheles*, a C-5 sterol desaturase and a histidine ammonia-lyase present in only *Tribolium *and two genes present in only honeybee, an ornithine carbamoyltransferase and a malonyl-CoA decarboxylase.

#### Novel case stories

Below we describe some of the identified losses that are likely to have had an impact on the functional divergence of the lineages, exemplifying losses of different types of orthologs, from the most conserved single-copy genes to orthologs with a highly patchy phylogenetic distribution. It has been suggested that secondary gene losses can be driven by the losses of key players of particular pathways or complexes that disable their functionality [25-27]. Hence, we mapped losses to the characterized biochemical pathways annotated for human orthologs; the results for *Tribolium *and its ancestor are overviewed in Table 1. However, having low numbers of losses per pathway [26], we concentrated more on providing examples of losses of directly interacting genes, reported for *Drosophila *from protein-protein interaction screens [28] and literature co-citations via human orthologs [29].

**Table 1:** Losses in *Tribolium *and its (Coleoptera/Diptera) ancestor mapped to pathways using human orthologs

Pathway	Source	Genes	Total	Only in beetle	Significance
Neuroactive ligand-receptor interaction	KEGG	*NPFFR1*, *NPFFR2*, *BZRP*, *TSPO*, *GALR1*, *GALR2*, *GALR3*	6	6	0.099
ABC transporters - general	KEGG	*ABCA1*, *ABCA4*, *ABCA12*, *ABCC5*, *ABCC12*	5	3	4.73E-005*
Oxidative phosphorylation	KEGG	*ATP6V0E*, *UCRC (7.2 kDa*), *NDUFA7*, *COX7C*	4	4	0.04
Cell cycle	KEGG	*CDC7*, *CCNE1*, *DBF4*	3	3	0.12
Folate biosynthesis/starch and sucrose metabolism	KEGG	*RAD54B*, *SETX*	2	2	0.06
Alkaloid biosynthesis II	KEGG	*DDHD1*, *SLC27A2*	2	0	0.02^†^
Regulation of actin cytoskeleton	KEGG	*FGD1*, *IQGAP1*	2	0	
Cholera - infection	KEGG	*ATP6V0E1*, *TRIM23*	2	1	0.06
Purine metabolism	KEGG	*PDE1C*, *POLR2L*	2	2	0.49
Ribosome	KEGG	*RPL29*, *RPL39*	2	2	0.53
Neurodegenerative disorders	KEGG	*BCL2L1*, *NGFR*	2	0	0.06
Propanoate metabolism	KEGG	*MLYCD*, *SLC27A2*	2	0	0.07
Methionine metabolism	KEGG	*DNMT1*, *DNMT3B*	2	0	
Oxidative stress induced gene expression via Nrf2	Biocarta	*HMOX1*, *NGFR*	2	1	0.02^†^

The statistical significance of the coordinated losses of at least two genes per pathway was calculated using hypergeometric test (* *p *< 0.01,^†^*p *< 0.05).

##### Losses of universal single-copy orthologs

An example of a universal single-copy ortholog missing in *Drosophila *is a 35 kDa protein associated with U11 snRNPs. U11 and U12 are components of the minor spliceosome responsible for the splicing of a small number of U12-type introns (<1% in both humans and flies) [30]. The minor spliceosome is widely conserved from plants to humans, including most insects but absent from *C. elegans*. Lack of a clear ortholog of U11 snRNA and the associated 35 kDa protein has been initially proposed [31], but Schneider *et al*. [32] identified a highly divergent U11 snRNA. The 31 kDa and 35 kDa proteins seem to be missing from all *Drosophila *species and a 25 kDa protein is absent from Diptera [33]. Interestingly, the loss of U11/U12 spliceosomal proteins in *Drosophila *is accompanied by the loss of the majority of U12-type introns [33,34].

Another example of a universal gene that seems to be missing from the *Drosophila *genome is sortilin-related receptor LR11 (also known as *SorLA*), a member of the low-density lipoprotein receptor family. LR11 binds low-density lipoprotein, the major cholesterol-carrying lipoprotein of plasma, and transports it into cells by endocytosis. Human LR11 also regulates trafficking of amyloid precursor protein and its expression is decreased in the brain of Alzheimer's disease patients [35].

##### Losses of universal multi-copy orthologs

An example is the *Cdc7 *kinase and its regulatory subunit *Dbf4 *implicated in triggering DNA replication in G1 phase through phosphorylation of Mcm proteins [36]. *Cdc7 *is essential in yeast in contrast to mice where homozygous null mutants for the *Cdc7 *ortholog *Nr2c2 *show impaired spermatogenesis [37]. *Cdc7 *is a universal single-copy gene with two fly paralogs whereas *Dbf4 *is present in two copies in humans and opossum. We confirmed the loss of both genes in *Tribolium *by tBlastn search and phylogenetic analysis (Additional data file 3). *Cdc7 *is also missing from the current *Anopheles *annotation but tBlastn searches identified a *Cdc7 *candidate in the genome. *Dbf4 *appears to be missing from the *Anopheles *and *Tetraodon *genomes. Interestingly, in yeast an allele of the gene *MCM5 *(*CDC46*) has been identified that bypasses the requirement for CDC7/DBF4 [38]. Although the *Tribolium *MCM5 ortholog TC_09146 does not feature the same mutation, P86L, it is conceivable that a similar mutation has rendered CDC7/DBF4 disposable in *Tribolium*.

Another example of a loss of an otherwise universal gene is the *Tribolium *ortholog of human ATP-binding cassette transporter A1 (ABCA1). ABCA1 is a cholesterol efflux transporter and is also required for engulfment of apoptotic cells by macrophages in mice and *C. elegans *[39,40]. In humans, the turnover of ABCA1 is regulated by Alpha1-syntrophin [41], and both genes encoding these proteins appear to be missing from the beetle genome.

##### Losses of patchy and insect-specific orthologs

We observed numerous losses in Diptera, many of which seem to be involved in the ubiquitin cycle, DNA repair (also reported in [31]), actin cytoskeleton and transcription control (Additional data file 4), which may point to substantial rearrangements of the ancestral pathways. An intriguing example is the BRCC complex, a complex with ubiquitin E3 ligase activity known to be involved in DNA repair, cell cycle regulation and homology-directed repair in human that has lost *BRCA1*, *RBBP-8 *and *BRCC3 *in the Dipteran lineage.

An example of insect-specific orthologous groups lost in Diptera are genes associated with oxidoreductase activity, including Aldo/keto reductases and several FAD dependent oxidoreductases; this category of genes was enriched among the 160 Diptera gene losses in a comparative Gene Ontology (GO) analysis with the *Tribolium *genome (Additional data file 5).

##### Extreme cases: exclusive insect models of human genes

At extremes, each novel insect genome sequence uncovers previously invisible orthologous gene relationships to human genes (see [10] for venn diagram that shows how many new orthologous relations are uncovered by the honeybee and beetle genomes). For example, we identified 45 orthologous groups shared between honeybee and at least one vertebrate but lost in the Coleoptera/Diptera ancestor, for example, an ortholog of RAD18 (GB-14468) that is an E3 ubiquitin-protein ligase involved in postreplication repair of UV-damaged DNA.

**Figure 4:**
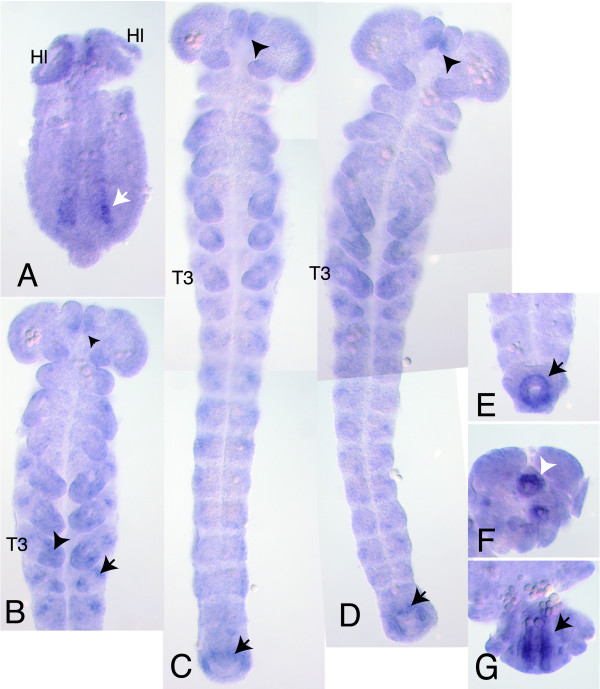
Expression pattern of the F-box gene during embryogenesis of the beetle *T. castaneum*. **(a) **F-box gene TC_04309 is initially expressed in the germ rudiment at the rims of the invaginating mesoderm, a position where activated Map-kinase is also seen [76]. Expression is strongest in the posterior (white arrow) and weakens towards the anterior. Ventral view, anterior is up posterior points down. Hl, head lobes. **(b) **Expression is seen as spots in the thoracic legs (arrowhead), at the base of the labral head appendages (small arrow head) and in segmentally repeated spots in the lateral body wall. T3, thoracic segment 3. Only the anterior half of the embryo is shown. **(c) **At a similar stage as shown in (b) where all body segments are present, the F-box gene is expressed in the anlagen of the hindgut (arrow). **(d) **When the legs have grown longer, F-box gene expression is extended covering the distal end. As seen in (c, d), expression in the labrum, in the hindgut-primordium and weakly at the lateral sites of the abdominal segments persists. **(e) **The hindgut has invaginated and grows out, forming a tube where the F-box gene is expressed in its posterior, proximal end around the future posterior gut opening (arrow). **(f) **At the retracted germ band stage, F-box is expressed around the anterior gut opening (white arrow) that has formed between the head lobes. **(g) **In the same embryo shown in (f), F-box gene expression is seen in the walls of the hindgut (arrow).

To complement the initial analysis of the *Tribolium *genome, we further identified 62 genes that are present in all vertebrates and *Tribolium *but lost from the other four insect genomes. Examples include *Yipf3*, a natural killer cell-specific antigen expressed during embryonic hematopoiesis in humans, and CENP-S, which in humans is a component of a centromeric protein complex, CENPA-CAD [42], that replaces histones in centromeres. In *Tribolium*, the orthologs of the other five complex members [42] seem to be absent from the genome, indicating a different mode of action. *CRLF3*, a cytokine receptor-like factor 3, has also been lost in all insects but *Tribolium*, as well as a regulator of the NF-κB pathway, *Tgf*, which positively regulates I-kappaB kinase. Because of structural and functional similarities in the mode of activation between insect and vertebrate NF-κB/Rel transcription factors, they are thought to have countered infections in Urbilateria [43]. Another *Tribolium *gene, TC_04309, absent in all sequenced arthropods, is an ortholog of the human F-box only gene 7 (*Fbxo7*). The tBlastn search and phylogenetic analysis of FBXO7 confirmed the lack of clear orthologs in other insects (Additional data files 6 and 7). FBXO7 is a component of modular E3 ubiquitin protein ligases called SCFs and functions in phosphorylation-dependent ubiquitination [44]. Human FBXO7 was also reported to positively regulate the activity of cyclin D/CDK6 in order to facilitate entry into the cell cycle [45]. In *Drosophila*, the cyclin D/CDK6 complex stimulates cell growth as well as proliferation [46,47]. Amniotes have two cdk4 orthologs due to a gene duplication (cdk4 and cdk6) and two cyclin D orthologs. *Tribolium *also encodes a cdk4 ortholog and two cyclin D orthologs, in contrast to the other insects, which have only one cyclin D ortholog (Additional data file 8). It is tempting to speculate that the presence of FBXO7 and a second cyclin D in the beetle are functionally linked. TC_04309 is expressed in limbs and the hindgut of *Tribolium *embryos (Figure 4). The function of TC_04309 is unknown but, taken together, it is conceivable that it controls cell proliferation in a tissue-specific manner as in mammals.

#### Robustness of estimates

Several factors can lead to an overestimation of gene losses: incomplete annotations, genome sequence gaps and the limited sensitivity of protein sequence comparison methods. Reassuringly, our estimates of number of gene losses for *Drosophila *and human, the two most extensively studied and curated model organisms, are similar to that of automatically annotated species, indicating a fairly good quality of genome annotations and their relative completeness. The chicken genome, however, shows exceptionally high numbers of losses in all categories that are likely to be overestimates due to an incomplete genome sequence that was estimated to be missing 5-10% of the genes [48], and, therefore, it was not taken into account in the analysis presented above. A closer inspection of 'missing' *Tribolium *genes from the universal single-copy and insect-specific fraction allowed us to correct about 30 *Tribolium *genes overlooked by the automatic annotation and some 150 merged genes. Nevertheless, most 'missing' genes were confirmed to be absent from the genome using tBlastn searches, indicating that the *Tribolium *annotation is nearly complete for evolutionarily conserved genes. Orthology misclassifications can also lead to inflated estimates when orthologous groups are wrongly split up, or to underestimates when several orthologous groups are spuriously pooled together, 'hiding' losses. We compared the results of our analysis with an independently derived and hand curated set of about 100 gene losses in Diptera (Hugh Robertson, personal communication). Detailed phylogenetic examination revealed only two to three cases of likely errors in our high throughput orthology identification pipeline and a few complicated cases that could not be resolved even using phylogenetic methods as the proteins were too short or too divergent.

## Discussion

We present here the quantification of losses of orthologous groups in five vertebrate and five insect genomes. Lineage-specific gene duplications result in multi-copy orthologous groups or fine-grained gene families. Here we focused on complete losses of such gene families (requiring all orthologous genes to be lost in a lineage). Members of an orthologous group are likely to share overlapping functions, and a complete loss of all representatives is more likely to have biological consequences [49,50] than a loss of a specific gene member. The parsimonious interpretation of the losses in the context of the species phylogeny suggests hundreds of gene losses on each branch of the tree. Diptera species lost the most genes, and placentals the least.

We show that the higher numbers of lost genes in insects can be explained by their higher rates of evolution as the loss rate is positively correlated with the molecular rate of evolution for each ortholog category and branch of the phylogeny. Interestingly, losses normalized for evolutionary rate and total number of orthologous groups are similar between insects and vertebrates, even for I and V orthologs. Therefore, one can not exclude that gene losses are mainly driven by neutral evolution [51,52], which should be taken as the null hypothesis until proven otherwise. Our data also suggest that about 20% of the gene repertoire evolves 8 times faster than the rest. The fact that the overall number of losses of orthologous groups is in agreement with the model of neutral evolution does not, of course, mean that all losses are selectively neutral. In that respect, it is noteworthy that some of the lost genes we discuss, such as *Cdc7/Dbf4 *or *Fboxo7*, seem to act as positive regulators.

Several hypotheses have been put forward to explain differences in evolutionary rates across species [53]. A high evolutionary rate might simply reflect differences in mutation rates. The known contributors to the rate of mutations, metabolic rate [54] and generation time [55], are clearly different between dipterans and mammals. In addition, differences in DNA methylation, fidelity of DNA-repair mechanisms or the production of DNA-damaging agents have also been suggested to explain different mutation rates in different species [53,56]. We found a number of genes implicated in DNA repair missing from Diptera. Although the functional consequences of these losses in Diptera are unknown, they might contribute to an increased mutation rate. A second hypothesis is that the efficiency of selection against deleterious mutants varies across species, due to differences in effective population size and/or mode of reproduction. Finally, rate variation across lineages could be caused by species-specific differences in the timing and frequency of adaptive evolution. Indeed, theoretical models [57,58] have proposed that evolvability is a selectable trait.

## Conclusion

We showed that the gene loss rate correlates well with rates of molecular evolution, explaining the significantly higher number of gene losses in insects. The data also can not reject that gene losses are dominated by neutral evolution.

The hundreds of lost genes we identified along the phylogenetic tree suggest common rearrangements and rewiring of ancient pathways and signaling cascades. Such global approaches are suitable for generating further experimentally testable hypotheses, and will lead to a better understanding of global evolutionary trends and detailed functional differences among lineages.

## Materials and methods

### Orthology classification

Protein sets were retrieved from Ensembl for *Drosophila*, *Anopheles *and all vertebrates as of 4 August 2006. *Tribolium *and *Apis *proteins were retrieved from Baylor College of Medecine and *Aedes *proteins from VectorBase. The assignment to orthologous groups was performed as described earlier [9,59,60]. Namely, we retained the longest open reading frame per locus and performed all-against-all comparisons using the Smith-Waterman algorithm as implemented in ParAlign [61] with default parameters. The orthologous groups were then assembled from the best reciprocal hits (BRHs; reciprocally best maching genes in between-genome-comparisons) applying a COG-like [62] procedure to join BRHs across three or more species, going from the best scoring ones until an E-value cut-off of 10^-6^, and keeping single BRH pairs only with E-values less than 10^-10^. Furthermore, the orthologous groups were expanded by genes that are more similar to each other within a proteome than to any gene in any of the other species, and by very similar copies that share over 97% sequence identity, which were identified initially using CD-hit [63]. All proteins in a group were required to have aligned regions overlapping by at least 20 residues to avoid the 'domain walking' effect.

### Species tree

A maximum likelihood species tree was calculated using the concatenated multiple alignments of 1,150 orthologs present in exactly one copy in all the organisms studied here. Multiple protein alignments were produced using muscle [64] and confidently aligned regions were extracted using Gblocks [65] with default settings. Individual protein alignments were concatenated into a 336,069 amino acid superalignment that was then subjected to maximum-likelihood analysis using the JTT model (G4+I+F) as implemented in PhyML [66] and we used Tree-Puzzle [67] to join separate bootstrap analyses. All shown branchings have at least 99% bootstrap support estimated from 500 replicates.

### Quantification of losses and correlation with other traits

Species radiation dates were taken from the literature: for insects from [18] and for vertebrates from [68,69]. We used MatLab version 7.2 (MathWorks, Natick MA, USA) for statistical analysis and data plotting. Regression lines were required to cross the origin. For each category of orthologs, the slopes of the regression lines for insects and vertebrates were compared based on a Student's *t*-distribution and were found not to be significantly different. Because traits were not normally distributed, we used non-parametric Spearman's correlation coefficients and Mann-Whitney U tests. Chicken data were excluded from graphs and statistical tests (see Discussion).

### Manual analysis of case studies

Selected orthologous groups were examined manually as follows. The absence of *Tribolium *proteins was verified by screening the *Tribolium *proteome, genome (assembled and single reads) and expressed sequence tags using the Baylor College of Medicine blast server [70]. All sufficiently similar sequences, including members of other orthologous groups, were aligned using muscle v3.6 [64] with default settings and all positions containing gaps were trimmed from conserved blocks using Gblocks [65]. Phylogenetic trees were constructed using maximum likelihood as implemented in PhyML [66] using the JTT model of amino acid substitution, a gamma distribution of rates over four rate categories and 100 bootstraps.

### Pathway mapping and database searches

We used pathway annotations from the KEGG database [71], mapping genes to Biocarta and co-citation analysis using Webgestalt [29] web interface. For data mining we used Ensembl [72], Swiss-prot/UniProt [73], Flybase [74], the Interactive Fly [22] and Online Mendelian Inheritance in Man [75] annotations.

## Abbreviations

ABCA1, ATP-binding cassette transporter A1; BRH, best reciprocal hit; CRLF3, cytokine receptor-like factor 3; GO, Gene Ontology; I, insect-specific orthologs; MYA, million years ago; N, universal multiple-copy orthologs; P, patchy orthologs; TERT, insect telomerase reverse transcriptase; U, universal single-copy orthologs; V, vertebrate-specific orthologs.

## Authors' contributions

SW and EMZ analyzed the data and wrote the manuscript. EK contributed the orthology data and the species phylogeny. RS and TK contributed experimental characterization of exclusive *Tribolium *and honeybee orthologs of human genes. EMZ supervised the project.

## Additional data files

The following additional data are available with the online version of this paper. Additional data file 1 is a graph showing the correlation between the absolute number of lost orthologous groups and and the rate of amino acid substitutions. Additional data files 2 and 3 provide the phylogenetic analysis of SID-1 and CDC7, respectively. Additional data file 4 is a table listing GO analysis of insect-specific orthologous groups lost in all Dipterans. Additional data file 5 is a table listing functionally linked genes coeliminated in the Diptera and Drosophila lineages. Additional file 6 provides the phylogenetic analysis of Fboxo7/cdk4/cyclin D. Additional data file 7 is a figure showing the alignment of Fboxo7 proteins.

## Supplementary Material

Additional data File 1 Correlation between the absolute number of lost orthologous groups and the rate of amino acid substitutions. Click here for file

Additional data File 2 Phylogenetic analysis of SID-1 proteins. Click here for file

Additional data File 3 Phylogenetic analysis of CDC7 proteins. Click here for file

Additional data File 4 GO analysis of insect-specific orthologous groups lost in all Dipterans. Click here for file

Additional data File 5 Coeliminiation of functionally linked genes in the Diptera and *Drosophila *lineages. Click here for file

Additional data File 6 Phylogenetic analysis of Fboxo7, cdk4 and cyclin D. Click here for file

Additional data File 7 Alignment of Fboxo7 proteins. Click here for file
