# Supplementary material for: Quantification of ortholog losses in insects and vertebrates
Source: Genome Biol. 2007 Nov 16;8(11):R242. doi: 10.1186/gb-2007-8-11-r242 (PMC2258195; doi:10.1186/gb-2007-8-11-r242)
Supplement: Additional data File 1 — Correlation between the absolute number of lost orthologous groups and the rate of amino acid substitutions. [file gb-2007-8-11-r242-S1.pdf]

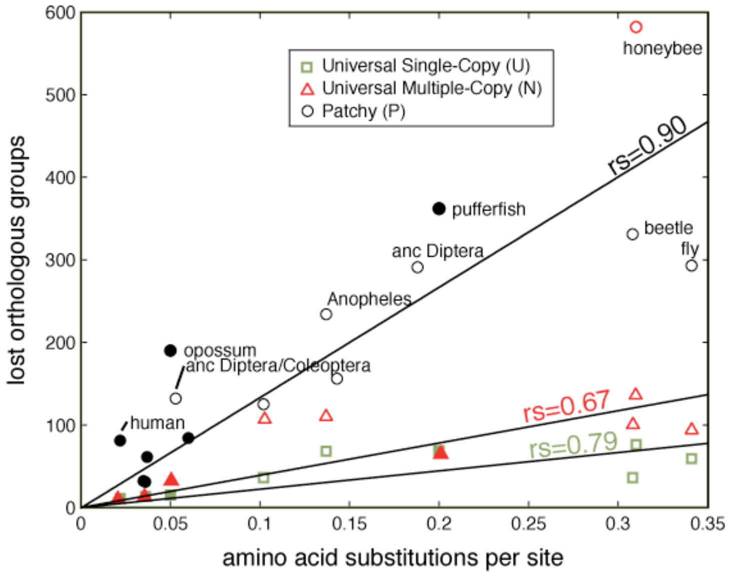

Additional data file 1. The number of lost orthologous groups correlates with the rate of amino acid substitutions.

The number of ancient orthologous groups (U, N, P) extinctions is plotted versus the branch length of the Maximum-Likelihood phylogenetic tree (see Figure 1). Filled symbols denote vertebrates and open symbols denote insects. Some selected losses of patchy orthologs are labelled. Abbreviations: anc – ancestral.
