# Supplementary material for: Quantification of ortholog losses in insects and vertebrates
Source: Genome Biol. 2007 Nov 16;8(11):R242. doi: 10.1186/gb-2007-8-11-r242 (PMC2258195; doi:10.1186/gb-2007-8-11-r242)
Supplement: Additional data File 2 — Phylogenetic analysis of SID-1 proteins. [file gb-2007-8-11-r242-S2.pdf]

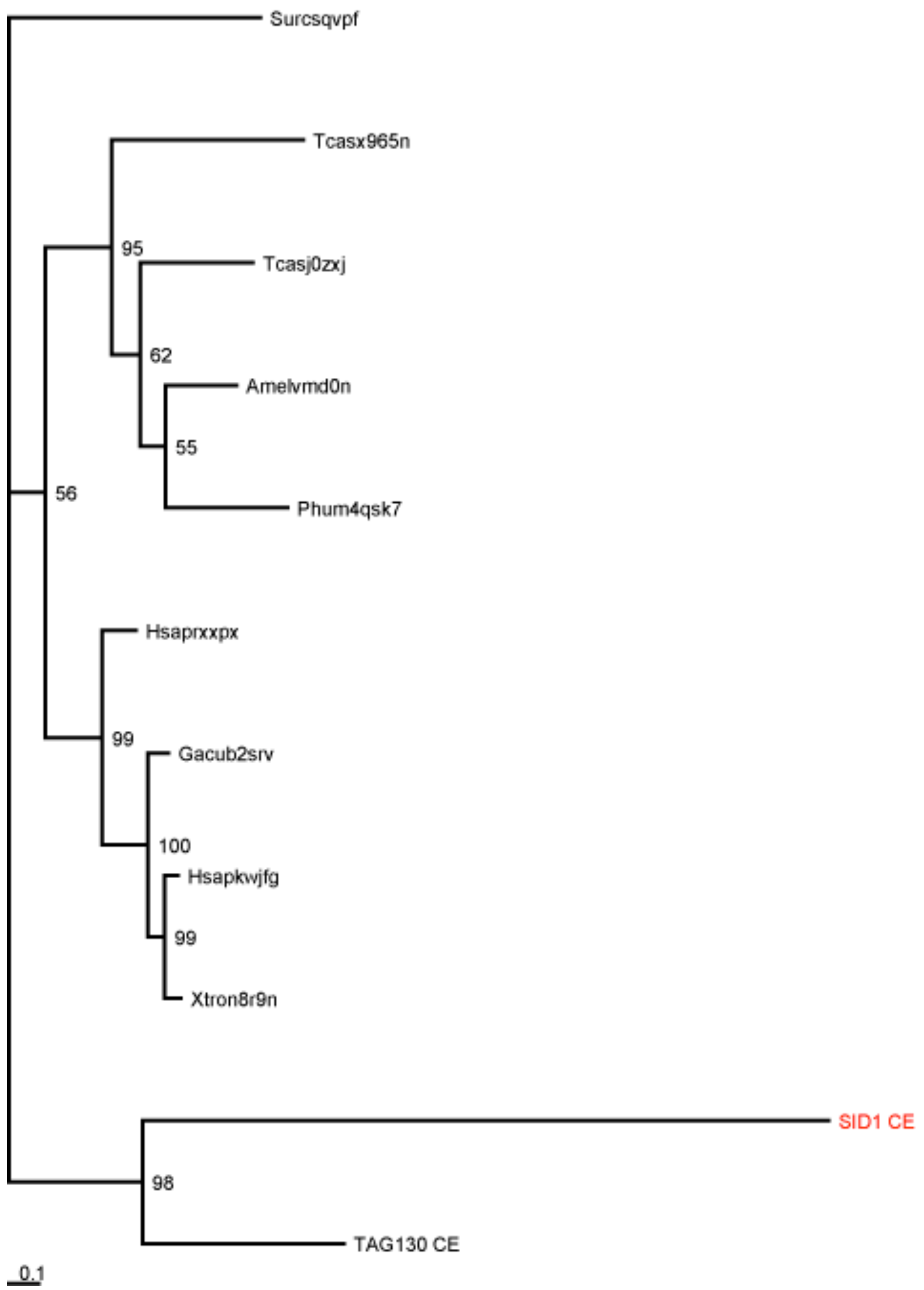

Additional data file 2. Maximum-likelihood phylogenetic analysis of SID-1 proteins. Branching confidence was estimated based on 100 bootstraps.
