# Supplementary material for: Quantification of ortholog losses in insects and vertebrates
Source: Genome Biol. 2007 Nov 16;8(11):R242. doi: 10.1186/gb-2007-8-11-r242 (PMC2258195; doi:10.1186/gb-2007-8-11-r242)
Supplement: Additional data File 3 — Phylogenetic analysis of CDC7 proteins. [file gb-2007-8-11-r242-S3.pdf]

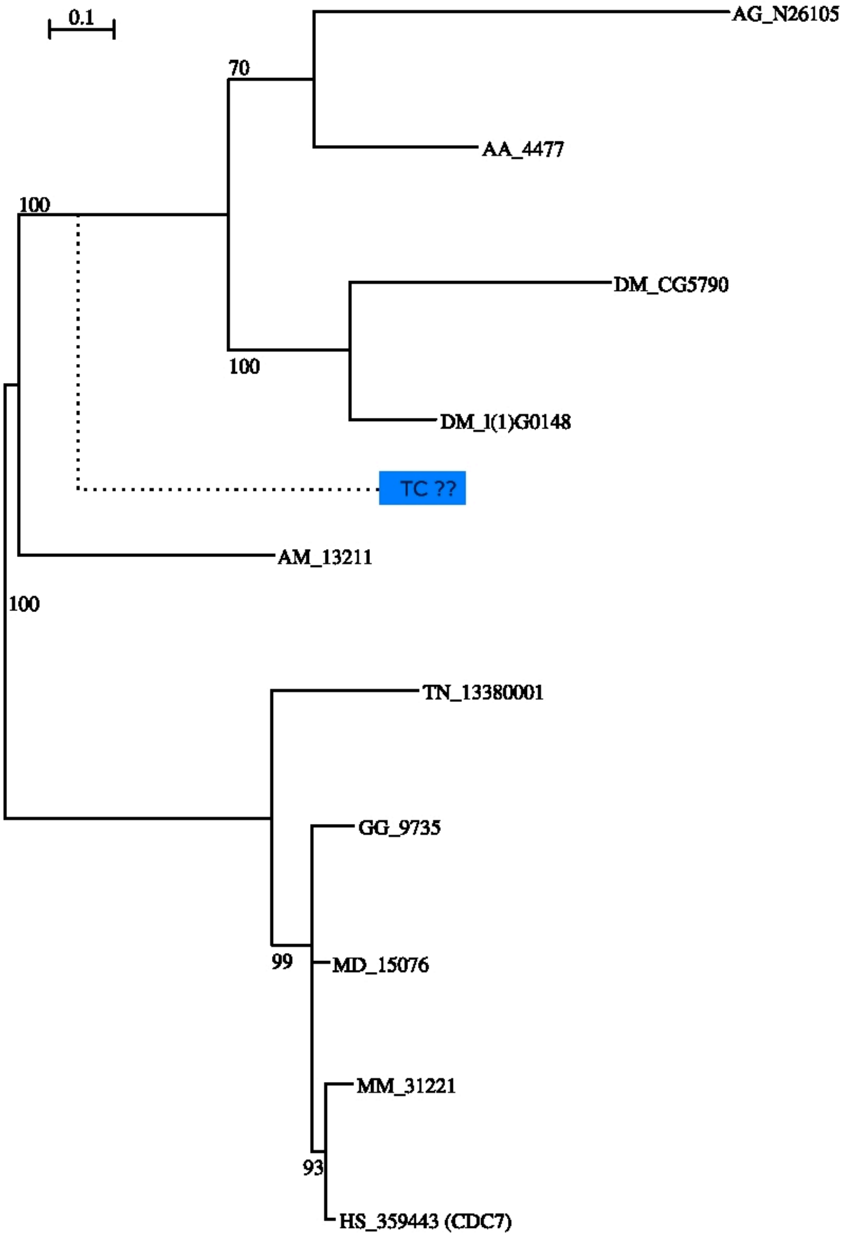

Additional data file 3. Maximum-likelihood phylogenetic analysis of CDC7 proteins. Branching confidence was estimated based on 100 bootstraps. Identifiers are ensembl protein ids preceded by a species abbreviation. CDC7 is present in all genomes except Tribolium. The Tribolium protein is missing from the genome, the closest Tribolium protein is TC_1853 which is the ortholog of casein kinase CSNK2A1/CNSKA2. As Anopheles CDC7 is not anotated in ensembl its annotation is from a homology-based gene annotation pipeline (http://cegg.unige.ch). DBF4 is fast evolving and the alignment is not sufficiently long for a phylogenetic tree.
