# Supplementary material for: Quantification of ortholog losses in insects and vertebrates
Source: Genome Biol. 2007 Nov 16;8(11):R242. doi: 10.1186/gb-2007-8-11-r242 (PMC2258195; doi:10.1186/gb-2007-8-11-r242)
Supplement: Additional data File 4 — GO analysis of insect-specific orthologous groups lost in all Dipterans. [file gb-2007-8-11-r242-S4.pdf]

Additional data file 4. GO terms which are under- or overrepresented in insect-specific orthologous groups lost in all Dipterans. Chi-Square Test without multiple testing correction. Significance levels are 0.05 (*) and <0.01 (**)

| Level | GO id | GO description | lost | present | lost % | present % | under/over-represented | Significance |
|---|---|---|---|---|---|---|---|---|
| **Molecular Function:** | | | | | | | | |
| catalytic activity | | | | | | | | |
| 3 | GO:0016491 | oxidoreductase activity | 16 | 449 | 11.9 | 4.2 | over | ** |
| **Biological Process:** | | | | | | | | |
| metabolism | | | | | | | | |
| 4 | GO:0009058 | biosynthesis | 1 | 458 | 0.7 | 4.2 | under | * |
| 4 | GO:0044238 | primary metabolism | 21 | 2592 | 15.7 | 24 | under | * |
| 5 | GO:0006139 | nucleobase, nucleoside, nucleot | 4 | 848 | 3 | 7.9 | under | * |
| **Cellular Component:** | | | | | | | | |
| 4 | GO:0005622 | intracellular | 11 | 1619 | 8.2 | 15 | under | * |
