# Supplementary material for: Quantification of ortholog losses in insects and vertebrates
Source: Genome Biol. 2007 Nov 16;8(11):R242. doi: 10.1186/gb-2007-8-11-r242 (PMC2258195; doi:10.1186/gb-2007-8-11-r242)
Supplement: Additional data File 5 — Coeliminiation of functionally linked genes in the Diptera and Drosophila lineages. [file gb-2007-8-11-r242-S5.pdf]

Additional data file 5. Coelimination of functionally linked genes in the Diptera and Drosophila lineages.

Protein function	Human Respresentati	Comments	ensembl id
Ubiquitin cycle	BRCA1	BREAST CANCER TYPE 1 SUSCEPTIBILITY HOMOLOG	ENSG00000012048
	BRCC3	BRCA1/BRCA2 CONTAINING COMPLEX SUBUNIT 3	ENSG00000185515
	UHRF1	UBIQUITIN PHD AND RING FINGER DOMAIN CONTAINING 2 EC_6.3.2.- UBIQUITIN CONTAINING PHD AND RIN	ENSG00000034063
	UBE2T	UBIQUITIN CONJUGATING ENZYME EC_6.3.2.19 UBIQUITIN LIGASE UBIQUITIN CARRIER	ENSG00000077152
	USP48	UBIQUITIN CARBOXYL TERMINAL HYDROLASE 48 EC_3.1.2.15 UBIQUITIN THIOESTERASE 48 UBIQUITIN S	ENSG00000090686
	MAP1LC3A	MICROTUBULE ASSOCIATED PROTEINS 1A/1B LIGHT CHAIN 3B MICROTUBULE ASSOCIATED 1 LIGHT CH	ENSG00000101460
	RNF8	UBIQUITIN LIGASE RNF8 EC_6.3.2.- RING FINGER 8	ENSG00000112130
	SUMO1	SMALL UBIQUITIN RELATED MODIFIER PRECURSOR SUMO UBIQUITIN SMT3 HOMOLOG	ENSG00000116030
	USPL1	AMBIGUOUS	ENSG00000132952
	FBXO6	F BOX ONLY	ENSG00000116663
	FBXO21	F BOX ONLY 21	ENSG00000135108
	RFWD2	RING FINGER AND WD REPEAT DOMAIN 2 EC_6.3.2.- UBIQUITIN LIGASE COP1 CONSTITUTIVE PHOTOMOR	ENSG00000143207
	BRE	BRE BRAIN AND REPRODUCTIVE ORGAN EXPRESSED	ENSG00000158019
	FBXO22	F BOX ONLY 22	ENSG00000167196
	UBE2J1	UBIQUITIN CONJUGATING ENZYME E2 6 EC_6.3.2.19 UBIQUITIN LIGASE UBC6 UBIQUITIN CARRIER UBC6	ENSG00000198833
	RNF19	RING FINGER 19	ENSG00000034677
	PCNP	PEST PROTEOLYTIC SIGNAL CONTAINING NUCLEAR PEST CONTAINING NUCLEAR PCNP	ENSG00000081154
	UFM1	UBIQUITIN FOLD MODIFIER 1 PRECURSOR	ENSG00000120686
	RBCK1	RANBP TYPE AND C3HC4 TYPE ZINC FINGER CONTAINING 1 UBIQUITIN CONJUGATING ENZYME 7 INTERAC	ENSG00000125826
	USP3	UBIQUITIN CARBOXYL TERMINAL HYDROLASE EC_3.1.2.15 UBIQUITIN THIOESTERASE UBIQUITIN SPECIFIC	ENSG00000140455
	ANAPC11	RING BOX 1	ENSG00000141552
	LRSAM1	UBIQUITIN LIGASE LRSAM1 EC_6.3.2.- LEUCINE RICH REPEAT AND STERILE ALPHA MOTIF CONTAINING 1	ENSG00000148356
	OTUD7B	OTU DOMAIN CONTAINING EC 3 ZINC FINGER CEZANNE	ENSG00000163113
DNA damage	COX19	CYTOCHROME C OXIDASE ASSEMBLY COX19	ENSG00000189201
	BRE	BRE BRAIN AND REPRODUCTIVE ORGAN EXPRESSED	ENSG00000158019
		AMBIGUOUS	ENSG00000162864
	BRCA1	BREAST CANCER TYPE 1 SUSCEPTIBILITY HOMOLOG	ENSG00000012048
	BRCC3	BRCA1/BRCA2 CONTAINING COMPLEX SUBUNIT 3	ENSG00000185515
	BRIP1	FANCONI ANEMIA GROUP J EC_3.6.1.- ATP DEPENDENT RNA HELICASE BRIP1 FACJ BRCA1 INTERACTIN	ENSG00000136492
	C14orf105	UNCHARACTERIZED	ENSG00000100557
	SIRT1	NAD DEPENDENT DEACETYLASE EC_3.5.1.-	ENSG00000096717
	BAG2	BAG FAMILY MOLECULAR CHAPERONE REGULATOR 2 BCL2 ASSOCIATED ATHANOGENE 2 BAG 2	ENSG00000112208
	CCDC53	COILED COIL DOMAIN CONTAINING 53	ENSG00000120860
	C12orf45	AMBIGUOUS	ENSG00000151131
	BHLHB5	OLIGODENDROCYTE TRANSCRIPTION FACTOR	ENSG00000180828
	PLEKHF2	PLECKSTRIN HOMOLOGY DOMAIN CONTAINING FAMILY F MEMBER PH DOMAIN CONTAINING FAMILY F ME	ENSG00000175895
	MED28	MEDIATOR OF RNA POLYMERASE II TRANSCRIPTION SUBUNIT 28 MEDIATOR COMPLEX SUBUNIT 28	ENSG00000118579
	STIM2	STROMAL INTERACTION MOLECULE PRECURSOR	ENSG00000109689
	SFRS14	SPLICING FACTOR ARGININE/SERINE RICH 14 ARGININE/SERINE RICH SPLICING FACTOR 14	ENSG00000064607
	SULT2B1	SULFOTRANSFERASE	ENSG00000088002
	FUT2	GALACTOSIDE 2 ALPHA L FUCOSYLTRANSFERASE 1 EC_2.4.1.69 GDP L FUCOSE:BETA D GALACTOSID	ENSG00000176920
		AMBIGUOUS	ENSG00000145835
Actin cytoskeleton	FGD1	FYVE RHOGEF AND PH DOMAIN CONTAINING ZINC FINGER FYVE DOMAIN CONTAINING	ENSG00000102302
	CORO2B	CORONIN	ENSG00000103647
	KPTN	KAPTIN	ENSG00000118162
	SDCBP	SYNTENIN SYNDECAN BINDING	ENSG00000137575
	KALRN	HUNTINGTIN ASSOCIATED INTERACTING PROTEIN DUO	ENSG00000160145
	DAPK1	MYOSIN LIGHT CHAIN KINASE MUSCLE EC_2.7.11.18	ENSG00000196730

Regulation of transcription regulation			
	BZRAP1	RIM BINDING RIM	ENSG00000005379
		JMJC DOMAIN CONTAINING HISTONE DEMETHYLATION EC_1.14.11.-	ENSG00000006459
	LGALS14	LECTIN	ENSG00000006659
	PGLYRP1	PEPTIDOGLYCAN RECOGNITION	ENSG00000008438
	BRCA1	BREAST CANCER TYPE 1 SUSCEPTIBILITY HOMOLOG	ENSG00000012048
	RNF14	RING FINGER 14 ANDROGEN RECEPTOR ASSOCIATED 54 TRIAD2	ENSG00000013561
	UHRF1	UBIQUITIN PHD AND RING FINGER DOMAIN CONTAINING 2 EC_6.3.2.- UBIQUITIN CONTAINING PHD AN	ENSG00000034063
	EPN3	EPSIN	ENSG00000049283
	SFRS14	SPLICING FACTOR ARGININE/SERINE RICH 14 ARGININE/SERINE RICH SPLICING FACTOR 14	ENSG00000064607
	ATXN3	ATAXIN 3 EC_3.4.22.- MACHADO JOSEPH DISEASE	ENSG00000066427
	KIF2A	KINESIN	ENSG00000068796
	CECR5	CAT EYE SYNDROME CRITICAL REGION 5 PRECURSOR	ENSG00000069998
	C14orf101	UNCHARACTERIZED	ENSG00000070269
	FOSL2	PROTO ONCOGENE C FOS CELLULAR ONCOGENE FOS	ENSG00000075426
	ARHGAP15	GTPASE ACTIVATING	ENSG00000075884
	FGF20	FIBROBLAST GROWTH FACTOR FGF	ENSG00000078579
	CHERP	AMBIGUOUS	ENSG00000085872
	SEPHS1	SELENIDE WATER DIKINASE EC_2.7.9.3 SELENOPHOSPHATE SYNTHETASE SELENIUM DONOR	ENSG00000086475
	NID2	NIDOGEN	ENSG00000087303
	SULT2B1	SULFOTRANSFERASE	ENSG00000088002
	VIL2	EZRIN 2	ENSG00000092820
	NUP188	UNKNOWN	ENSG00000095319
	SIRT1	NAD DEPENDENT DEACETYLASE EC_3.5.1.-	ENSG00000096717
	NDUFB7	NADH DEHYDROGENASE [UBIQUINONE] 1 BETA SUBCOMPLEX SUBUNIT 7 EC_1.6.5.3 EC_1.6.99.- 3	ENSG00000099795
	GPSN2	SYNAPTIC GLYCOPROTEIN SC2	ENSG00000099797
	GADD45B	GROWTH ARREST AND DNA DAMAGE INDUCIBLE GADD45	ENSG00000099860
	C14orf105	UNCHARACTERIZED	ENSG00000100557
	ADNP	ACTIVITY DEPENDENT NEUROPROTECTOR ACTIVITY DEPENDENT NEUROPROTECTIVE	ENSG00000101126
	HRH3	MUSCARINIC ACETYLCHOLINE RECEPTOR	ENSG00000101180
	BPI	LIPOPOLYSACCHARIDE BINDING PRECURSOR LBP	ENSG00000101425
	ARHGEF7	RHO GUANINE NUCLEOTIDE EXCHANGE FACTOR 6 RAC/CDC42 GUANINE NUCLEOTIDE EXCHANGE FA	ENSG00000102606
	NUP93	NUCLEAR PORE COMPLEX NUP93 NUCLEOPORIN NUP93.93 KDA NUCLEOPORIN	ENSG00000102900
	GSPT1	EUKARYOTIC PEPTIDE CHAIN RELEASE FACTOR GTP BINDING SUBUNIT ERF2 TRANSLATION RELEASE FACT	ENSG00000103342
	BFAR	BIFUNCTIONAL APOPTOSIS REGULATOR	ENSG00000103429
	NKG7	NKG7 NATURAL KILLER CELL 7	ENSG00000105374
	KIAA0241	AMBIGUOUS	ENSG00000105778
	SPIN	SPINDLIN	ENSG00000106723
	LHX6	LIM/HOMEOBOX	ENSG00000106852
	PLEKHA1	PLECKSTRIN HOMOLOGY DOMAIN CONTAINING FAMILY A MEMBER 1 TANDEM PH DOMAIN CONTAINING	ENSG00000107679
	TCF2	HEPATOCYTE NUCLEAR FACTOR 1 HNF	ENSG00000108753
	PMP22	EPITHELIAL MEMBRANE EMP	ENSG00000109099
	STIM2	STROMAL INTERACTION MOLECULE PRECURSOR	ENSG00000109689
	PPARGC1A	PEROXISOME PROLIFERATOR ACTIVATED RECEPTOR GAMMA COACTIVATOR 1 ALPHA PPAR GAMMA CO	ENSG00000109819
	ELP4	AMBIGUOUS	ENSG00000109911
	CEP164	UNKNOWN	ENSG00000110274
	ART4	ECTO ADP RIBOSYLTRANSFERASE PRECURSOR EC_2.4.2.31 NAD P + ARGININE ADP RIBOSYLTRANSFERASE	ENSG00000111339
	MGP	MATRIX GLA PRECURSOR MGP	ENSG00000111341
	C6orf105	UNCHARACTERIZED	ENSG00000111863
	MAN1A1	MANNOSYL OLIGOSACCHARIDE 1 2 ALPHA MANNOSIDASE EC_3.2.1.113 PROCESSING ALPHA 1 2 MANN	ENSG00000111885
	BAG2	BAG FAMILY MOLECULAR CHAPERONE REGULATOR 2 BCL2 ASSOCIATED ATHANOGENE 2 BAG 2	ENSG00000112208
	PRDM13	PR DOMAIN ZINC FINGER 13 PR DOMAIN CONTAINING 13	ENSG00000112238
	EIF1B	EUKARYOTIC TRANSLATION INITIATION FACTOR TRANSLATION FACTOR SUI1 HOMOLOG	ENSG00000114784
	HSPE1	10 KDA HEAT SHOCK PROTEIN MITOCHONDRIAL HSP10.10 KDA CHAPERONIN	ENSG00000115541
	SMYD1	SET AND MYND DOMAIN CONTAINING 3 EC_2.1.1.43 ZINC FINGER MYND DOMAIN CONTAINING 1	ENSG00000115593
	SUMO1 / SUMO1P3	SMALL UBIQUITIN RELATED MODIFIER PRECURSOR SUMO UBIQUITIN SMT3 HOMOLOG	ENSG00000116030
	CD46	MEMBRANE COFACTOR PRECURSOR CD46 ANTIGEN	ENSG00000117335
	MED28	MEDIATOR OF RNA POLYMERASE II TRANSCRIPTION SUBUNIT 28 MEDIATOR COMPLEX SUBUNIT 28	ENSG00000118579
	MSX2	HOMEOBOX MSX 1 MSH HOMEOBOX 1	ENSG00000120149
	EXOSC8	EXOSOME COMPLEX EXONUCLEASE RRP43 EC_3.1.13.- RIBOSOMAL RNA PROCESSING 43 EXOSOME COMP	ENSG00000120699
	CCDC53	COILED COIL DOMAIN CONTAINING 53	ENSG00000120860
	TMEM39B	AMBIGUOUS	ENSG00000121775
	PLG	PLASMINOGEN EC_3.4.21.7 [CONTAINS: PLASMIN HEAVY CHAIN A; PLASMIN LIGHT CHAIN B]	ENSG00000122194
	TWIST1	TWIST RELATED	ENSG00000122691
	CSE1L	EXPORTIN 2 EXP2 IMPORTIN ALPHA RE EXPORTER CHROMOSOME SEGREGATION 1	ENSG00000124207
	SIRT5	NAD DEPENDENT DEACETYLASE SIRTUIN 5 EC_3.5.1.- SIR2 5	ENSG00000124523
	PRMT1	ARGININE METHYLTRANSFERASE EC_2.1.1.-	ENSG00000126457
	IL22	INTERLEUKIN PRECURSOR IL IL 10 RELATED T CELL DERIVED INDUCIBLE FACTOR IL TIF	ENSG00000127318
	DOCK6	DEDICATOR OF CYTOKINESIS	ENSG00000130158
		EXOCYST COMPLEX COMPONENT 3 EXOCYST COMPLEX COMPONENT SEC6	ENSG00000130201
	PVRL2	POLIOVIRUS RECEPTOR RELATED PRECURSOR HERPES VIRUS ENTRY MEDIATOR NECTIN ANTIGEN	ENSG00000130202
	APOE	APOLIPOPROTEIN E APO E	ENSG00000130203
	TOMM40	PROBABLE MITOCHONDRIAL IMPORT RECEPTOR SUBUNIT TOM40 HOMOLOG TRANSLOCASE OF OUTER	ENSG00000130204
	APOC2 APOC4	APOLIPOPROTEIN C IV PRECURSOR APO CIV APOC IV	ENSG00000130207
	DNMT1	DNA CYTOSINE 5 METHYLTRANSFERASE EC_2.1.1.37 DNMT1 DNA METHYLTRANSFERASE DNA MTASE	ENSG00000130816
	ZNF341	ZINC FINGER 341	ENSG00000131061
	C1QL1	C1Q RELATED FACTOR PRECURSOR COMPLEMENT COMPONENT 1 Q SUBCOMPONENT 1	ENSG00000131094
		SWITCH ASSOCIATED 70 SWAP 70	ENSG00000133789
	MICALCL	AMBIGUOUS	ENSG00000133808
	MICAL2	MICAL	ENSG00000133816
	RINT1	RAD50 INTERACTING 1 RAD50 INTERACTOR 1 RINT 1	ENSG00000135249
	C13orf34	AMBIGUOUS	ENSG00000136122
	RCBTB1	RCC1 AND BTB DOMAIN CONTAINING 2 REGULATOR OF CHROMOSOME CONDENSATION AND BTB D	ENSG00000136144
	CIB2	CALCIUM AND INTEGRIN BINDING 1 CALMYRIN DNA PKCS INTERACTING KINASE INTERACTING KIP CIB	ENSG00000136425
	BRIP1	FANCONI ANEMIA GROUP J EC_3.6.1.- ATP DEPENDENT RNA HELICASE BRIP1 FACJ BRCA1 INTERACTI	ENSG00000136492
	TFAP2A	TRANSCRIPTION FACTOR AP 2 ALPHA AP2 ALPHA ACTIVATING ENHANCER BINDING 2 ALPHA ACTIVA	ENSG00000137203
	TMEM180	UNCHARACTERIZED MEMBRANE	ENSG00000138111
	LBX1	TRANSCRIPTION FACTOR LBX1	ENSG00000138136
	SEPT11	SEPTIN	ENSG00000138758
	WDR89	WD REPEAT	ENSG00000140006
	RANBP10	RAN BINDING 9 RANBP9 RAN BINDING M RANBPM	ENSG00000141084
	RFX5	DNA BINDING RFX5 REGULATORY FACTOR X SUBUNIT 5	ENSG00000143390
	VGLL4	TRANSCRIPTION COFACTOR VESTIGIAL 4 VGL 4	ENSG00000144560
	EAF1	ELL ASSOCIATED FACTOR 2 TESTOSTERONE REGULATED APOPTOSIS INDUCER AND TUMOR SUPPRESSOR	ENSG00000144597

LHFPL2	LIPOMA HMGIC FUSION PARTNER 2	ENSG00000145685
	AMBIGUOUS	ENSG00000145835
IRAK1BP1	CDNA PRODUCT:ACTA BINDING 3	ENSG00000146243
ATP6V1B2	VACUOLAR ATP SYNTHASE SUBUNIT B EC_3.6.3.14 V ATPASE SUBUNIT VACUOLAR PROTON PUMP E	ENSG00000147416
UHRF2	UBIQUITIN PHD AND RING FINGER DOMAIN CONTAINING 2 EC_6.3.2.- UBIQUITIN CONTAINING PHD ANI	ENSG00000147854
KIRREL3	KIN OF IRRE PRECURSOR KIN OF IRREGULAR CHIASM NEPHRIN	ENSG00000149571
EVA1	1 PRECURSOR	ENSG00000149573
C12orf45	AMBIGUOUS	ENSG00000151131
PSTPIP2	PROLINE SERINE THREONINE PHOSPHATASE INTERACTING	ENSG00000152229
TRIM11	TRIPARTITE MOTIF CONTAINING	ENSG00000154370
DCK	KINASE	ENSG00000156136
	ANKYRIN	ENSG00000157999
MRPL17	39S RIBOSOMAL L17 MITOCHONDRIAL PRECURSOR L17MT MRP L17	ENSG00000158042
TMSL8	ADULT MALE TESTIS CDNA PRODUCT:THYMOSIN BETA HOMOLOG E11 GENE	ENSG00000158164
HIST1H2BD	HISTONE H2B	ENSG00000158373
HIST1H4H	HISTONE H4	ENSG00000158406
MITD1	AMBIGUOUS	ENSG00000158411
EIF5B	EUKARYOTIC TRANSLATION INITIATION FACTOR 5B EIF 5B TRANSLATION INITIATION FACTOR IF 2	ENSG00000158417
	AMBIGUOUS	ENSG00000158428
C2orf29	UNKNOWN	ENSG00000158435
	AMBIGUOUS	ENSG00000159374
PSMB4	PROTEASOME SUBUNIT BETA TYPE 4 PRECURSOR EC_3.4.25.1 PROTEASOME BETA CHAIN MACROPAINE	ENSG00000159377
IRX6	IROQUOIS CLASS HOMEODOMAIN IRX IROQUOIS HOMEOBOX HOMEODOMAIN	ENSG00000159387
CES7	LIVER CARBOXYLESTERASE PRECURSOR EC_3.1.1.1	ENSG00000159398
HK2	HEXOKINASE EC_2.7.1.1	ENSG00000159399
	COMPLEMENT SUBCOMPONENT PRECURSOR [CONTAINS: COMPLEMENT SUBCOMPONENT HEAVY CHAIN	ENSG00000159403
ASRGL1	THREONINE ASPARTASE 1 EC_3.4.25.- TASPASE 1 [CONTAINS: THREONINE ASPARTASE SUBUNIT ALPI	ENSG00000162174
LRRC38	LEUCINE RICH REPEAT CONTAINING	ENSG00000162494
SNED1	PRECURSOR	ENSG00000162804
	AMBIGUOUS	ENSG00000162864
REL	NUCLEAR FACTOR NF KAPPA B SUBUNIT	ENSG00000162924
PEX13	PEROXISOMAL MEMBRANE PEX13 PEROXIN 13	ENSG00000162928
RFTN2	RAFTLIN 2 RAFT LINKING 2	ENSG00000162944
CAPN13	CALPAIN SUBUNIT CALPAIN SUBUNIT CALCIUM ACTIVATED NEUTRAL PROTEINASE CANP	ENSG00000162949
LRRTM1	LEUCINE RICH REPEAT TRANSMEMBRANE NEURONAL PRECURSOR	ENSG00000162951
C2orf47	UNKNOWN	ENSG00000162972
ARL5A	ADP RIBOSYLATION FACTOR	ENSG00000162980
FABP1	FATTY ACID BINDING PROTEIN LIVER L FABP	ENSG00000163586
PPM1L	PHOSPHATASE 2C EC_3.1.3.16 PP2C	ENSG00000163590
SFMBT1	SCM WITH FOUR MBT DOMAINS	ENSG00000163935
CCDC112	AMBIGUOUS	ENSG00000164221
OXR1	OXIDATION RESISTANCE 1	ENSG00000164830
ATP5C1	ATP SYNTHASE GAMMA CHAIN MITOCHONDRIAL PRECURSOR EC_3.6.3.14	ENSG00000165629
CD300C	CMRF35 MOLECULE 1 PRECURSOR CLM 1 CD300 ANTIGEN FAMILY MEMBER F	ENSG00000167850
DTYMK	THYMIDYLATE KINASE EC_2.7.4.9 DTMP KINASE	ENSG00000168393
RAB24 / MXD3	RAS RELATED RAB	ENSG00000169228
KLF13	KRUEPPEL FACTOR TRANSCRIPTION FACTOR BASIC TRANSCRIPTION ELEMENT BINDING BTE BINDING	ENSG00000169926
	LECTIN	ENSG00000170298
PRDM10	PR DOMAIN ZINC FINGER 10 PR DOMAIN CONTAINING 10	ENSG00000170325
GATM	GLYCINE AMIDINOTRANSFERASE MITOCHONDRIAL PRECURSOR EC_2.1.4.1 L ARGININE:GLYCINE AMIDINO	ENSG00000171766
	LRP16	ENSG00000172264
HPSE2	HEPARANASE PRECURSOR EC_3.2.-.- ENDO GLUCORONIDASE [CONTAINS: HEPARANASE 8 KDA SUBUN	ENSG00000172987
GOLPH4	130 GOLGI LOCALIZED PHOSPHOPROTEIN	ENSG00000173905
GOLT1A	VESICLE TRANSPORT GOLGI TRANSPORT 1 HOMOLOG	ENSG00000174567
INHBC	INHIBIN BETA CHAIN PRECURSOR ACTIVIN BETA CHAIN	ENSG00000175189
PLEKHF2	PLECKSTRIN HOMOLOGY DOMAIN CONTAINING FAMILY F MEMBER PH DOMAIN CONTAINING FAMILY F ME	ENSG00000175895
OR4D11	OLFACTORY RECEPTOR	ENSG00000176200
FUT2	GALACTOSIDE 2 ALPHA L FUCOSYLTRANSFERASE 1 EC_2.4.1.69 GDP L FUCOSE:BETA D GALACTOSIDE	ENSG00000176920
NFATC2IP	FACTOR OF ACTIVATED T CELLS CYTOPLASMIC 2 INTERACTING	ENSG00000176953
KIAA0195	TRANSMEMBRANE 94	ENSG00000177728
	RNA POLYMERASE II TRANSCRIPTIONAL COACTIVATOR	ENSG00000177907
ASB8	ANKYRIN REPEAT AND SOCS BOX 8 ASB 8	ENSG00000177981
MPI	MANNOSE 6 PHOSPHATE ISOMERASE EC_5.3.1.8 PHOSPHOMANNOSE ISOMERASE PMI PHOSPHOHEXOMUT	ENSG00000178802
C9orf50	AMBIGUOUS	ENSG00000179058
	OLFACTORY RECEPTOR	ENSG00000180144
RRH	OPSIN	ENSG00000180245
TMEM64	UNKNOWN	ENSG00000180694
BHLHB5	OLIGODENDROCYTE TRANSCRIPTION FACTOR	ENSG00000180828
TDRKH	TUDOR AND KH DOMAIN CONTAINING	ENSG00000182134
AFMID	PROBABLE ARYLFORMAMIDASE EC_3.5.1.9 KYNURENINE FORMAMIDASE KF	ENSG00000183077
OR2T11	OLFACTORY RECEPTOR OLFACTORY RECEPTOR OR1	ENSG00000183130
	UNCHARACTERIZED	ENSG00000183260
GSTT1	GLUTATHIONE S TRANSFERASE THETA EC_2.5.1.18 GST CLASS THETA	ENSG00000184674
LCN12	EPIDIDYMAL SPECIFIC LIPOCALIN 12 PRECURSOR	ENSG00000184925
C9orf18	AMBIGUOUS	ENSG00000185681
OR4C46	OLFACTORY RECEPTOR OLFACTORY RECEPTOR OR11	ENSG00000185926
	60S RIBOSOMAL	ENSG00000186239
MYT1L	MYELIN TRANSCRIPTION FACTOR 1	ENSG00000186487
	AMBIGUOUS	ENSG00000186871
	14 DAYS EMBRYO LIVER CDNA PRODUCT: FRAGMENT	ENSG00000187867
COX19	CYTOCHROME C OXIDASE ASSEMBLY COX19	ENSG00000189201
PNRC2	PROLINE RICH NUCLEAR RECEPTOR COACTIVATOR 2	ENSG00000189266
C8orf76	UNCHARACTERIZED	ENSG00000189376
	UNKNOWN	ENSG00000197980
CCDC16	COILED COIL DOMAIN CONTAINING 16	ENSG00000198783
OR5D13	OLFACTORY RECEPTOR	ENSG00000198877
SP5	TRANSCRIPTION FACTOR	ENSG00000204335
	AMBIGUOUS	ENSG00000204676
	TESTIS EXPRESSED 101 PRECURSOR LIPID RAFT ASSOCIATED GLYCOPROTEIN TEC 21	ENSG00000204933
OR6C1	OLFACTORY RECEPTOR	ENSG00000205330
	UNKNOWN	ENSG00000205765
