# Supplementary material for: Quantification of ortholog losses in insects and vertebrates
Source: Genome Biol. 2007 Nov 16;8(11):R242. doi: 10.1186/gb-2007-8-11-r242 (PMC2258195; doi:10.1186/gb-2007-8-11-r242)
Supplement: Additional data File 6 — Phylogenetic analysis of Fboxo7, cdk4 and cyclin D. [file gb-2007-8-11-r242-S6.pdf]

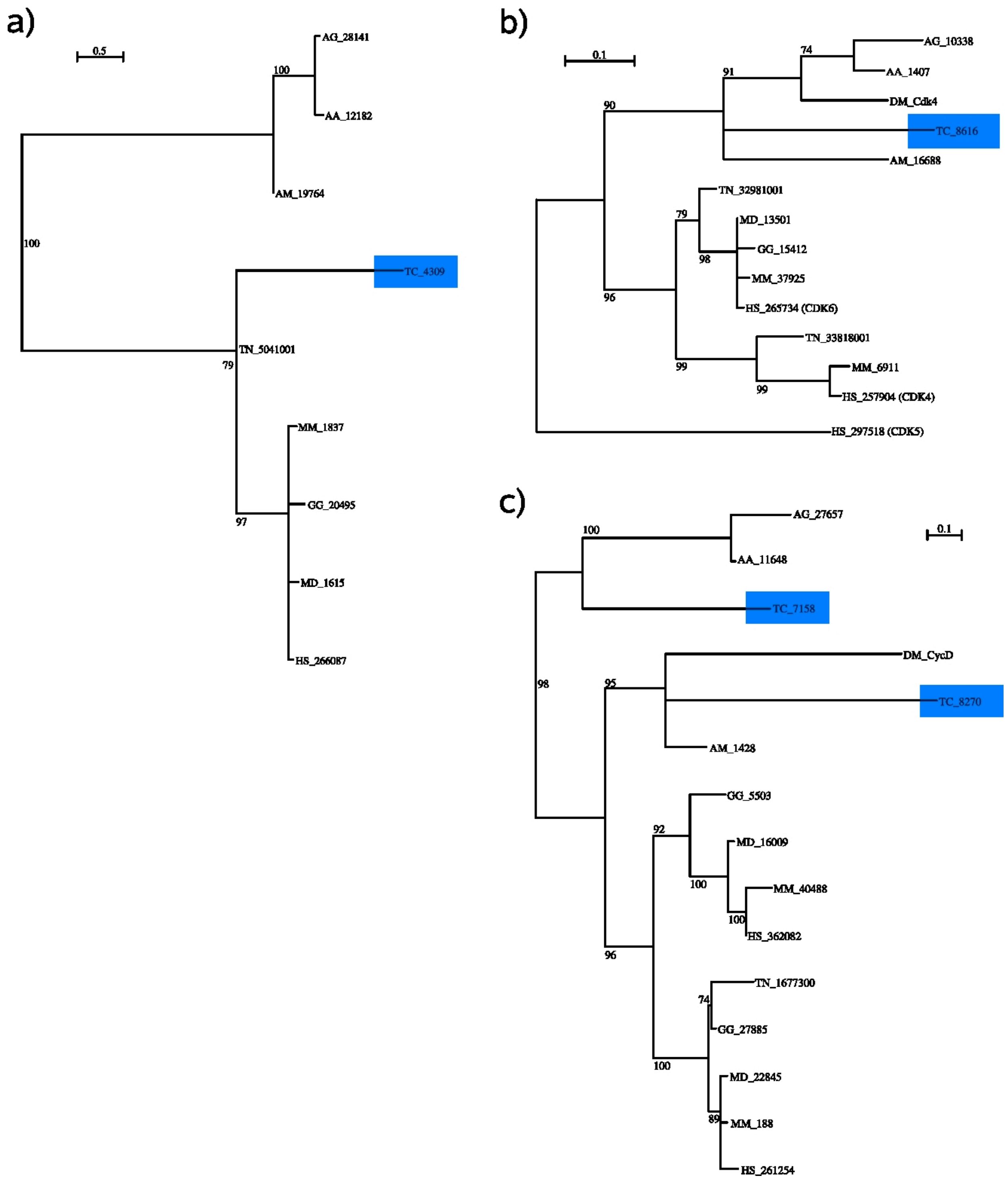

Additional data file 6. Phylogenetic analysis of Fbox7 (a), cdk4 (b) and cyclin D (c). Maximum-likelihood trees were calculated using phyml. Branchings with less than 70% bootstrap support (100 bootstraps) were collapsed. Tribolium proteins are underlayed in blue. (a) Fbox7 is divergent in diptera and missing from Drosophila, whereas the Tribolium ortholog is vertebrate-like. The closest blast hit of human Fbx07 to Drosophila is Pi4KIIalpha (CG2929) which is the Drosophila ortholog of phosphatidylinositol 4-kinase type II (PI4K2B). Interestingly, there is no obvious Fbx07 ortholog in Ciona or c. elegans.
