# Supplementary material for: Quantification of ortholog losses in insects and vertebrates
Source: Genome Biol. 2007 Nov 16;8(11):R242. doi: 10.1186/gb-2007-8-11-r242 (PMC2258195; doi:10.1186/gb-2007-8-11-r242)
Supplement: Additional data File 7 — Alignment of Fboxo7 proteins. [file gb-2007-8-11-r242-S7.pdf]

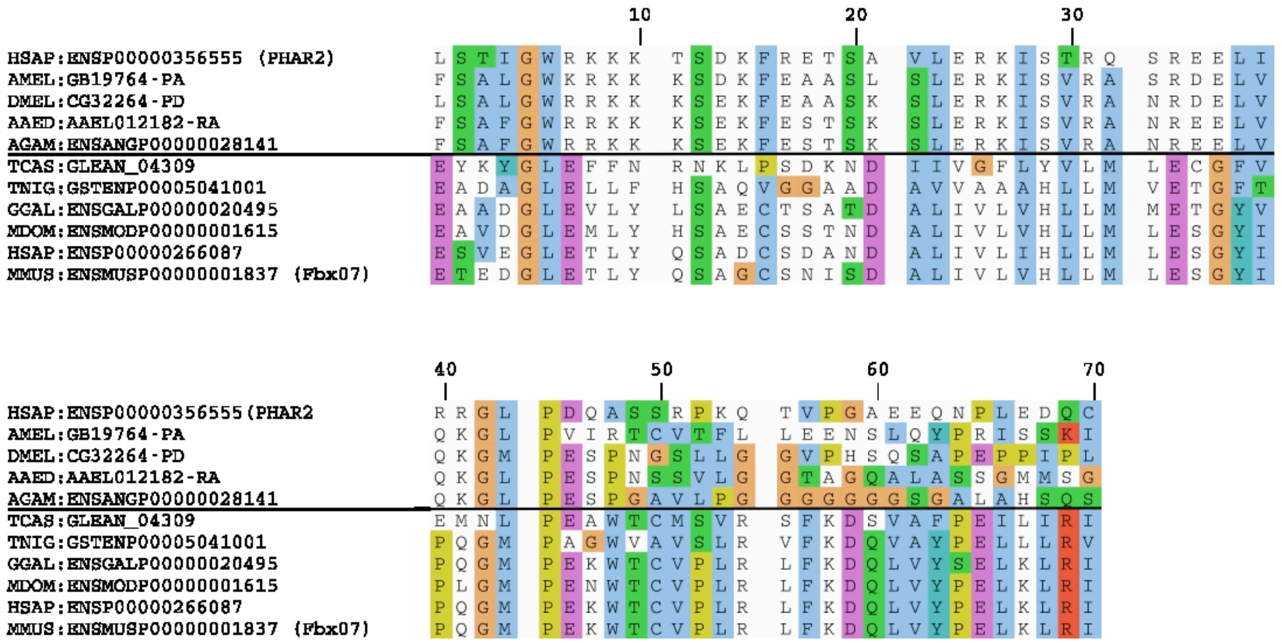

Additional data file 7. Alignment of Fbox7 proteins (below the black line) and Phosphatase and actin regulators PHAR2 (above the black line). Tribolium Glean_04309 is more similar to vertebrate Fbox7 proteins than to insect proteins.
